# Risk Factors for Early-Onset Peripheral Neuropathy Caused by Vincristine in Patients With a First Administration of R-CHOP or R-CHOP-Like Chemotherapy

**DOI:** 10.14740/jocmr1856w

**Published:** 2014-05-22

**Authors:** Naoto Okada, Takeshi Hanafusa, Takumi Sakurada, Kazuhiko Teraoka, Toshihide Kujime, Masahiro Abe, Yasuo Shinohara, Kazuyoshi Kawazoe, Kazuo Minakuchi

**Affiliations:** a Department of Pharmacy, Tokushima University Hospital, 2-50-1 Kuramoto, Tokushima 770-8503, Japan; b Faculty of Pharmaceutical Sciences, University of Tokushima, Shomachi-1, Tokushima 770-8505, Japan; c Department of Medicine and Bioregulatory Sciences, Graduate School of Medical Sciences, University of Tokushima, 3-8-15 Kuramoto, Tokushima 770-8503, Japan; d Institute for Genome Research, University of Tokushima, 3 Kuramoto, Tokushima 770-8503, Japan; e Department of Clinical Pharmacy, Institute of Health Biosciences, University of Tokushima, 2-50-1 Kuramoto, Tokushima 770-8503, Japan

**Keywords:** Vincristine, Early-onset peripheral neuropathy, Risk factor, Aprepitant, Dose of vincristine

## Abstract

**Background:**

Peripheral neuropathy is a well-known side effect of vincristine (VCR), a microtubule inhibitor used for R-CHOP or R-CHOP-like (namely R-CVP and R-THP-COP) regimens. Previous studies have shown that both the total dose of VCR and the number of treatment cycles are related to the incidence of VCR-induced peripheral neuropathy (VIPN). However, VIPN will also occur during the first treatment cycle regardless of the total dose of VCR or number of treatment cycles (early-onset VIPN). There is little information about early-onset VIPN, and it is difficult to predict. The present study’s goal was to identify risk factors for early-onset VIPN.

**Methods:**

We analyzed the case records of patients who had their first administration of an R-CHOP or R-CHOP-like regimen between April 2008 and August 2013 at Tokushima University Hospital in Tokushima, Japan. To identify the risk factors for early-onset VIPN, we performed univariate and multivariate logistic regression analyses.

**Results:**

Forty-one patients underwent an R-CHOP or R-CHOP-like regimen for the first time at Tokushima University Hospital between April 2008 and August 2013, and 14 patients had grade 1 or higher early-onset VIPN. A univariate analysis revealed that age, the dose of VCR and the concomitant use of aprepitant appeared to be the risk factors of early-onset VIPN. In our calculation using receiver-operator characteristics curves, the cut-off value for patient age was 65 years and that of the dose of VCR was 1.9 mg. A multivariate analysis revealed that VCR dose ≥ 1.9 mg and the concomitant use of the antiemetic aprepitant were independent risk factors for early-onset VIPN.

**Conclusions:**

Our present study showed that the patients who had VCR dose ≥ 1.9 mg and the concomitant use of aprepitant had the risk for early-onset VIPN. This suggests that it is important to use aprepitant in light of the risk of early-onset VIPN and the benefit of aprepitant’s antiemetic effect in R-CHOP and R-CHOP-like regimens.

## Introduction

Vincristine (VCR), a microtubule assembly inhibitor, is a key drug for the treatment of B-cell lymphoma [1]. VCR-based chemotherapies such as R-CHOP (rituximab, cyclophosphamide, adriamycin, vincristine and prednisolone), R-CVP (rituximab, cyclophosphamide, vincristine and prednisolone) and R-THP-COP (rituximab, cyclophosphamide, tetrahydropyranyladriamycin, vincristine and prednisolone) are standard regimens in the treatment of B-cell lymphoma [2-4]. One of the most common adverse events of R-CHOP and R-CHOP-like regimens (namely R-CVP and R-THP-COP) is VCR-induced peripheral neuropathy (VIPN). Previous studies have shown that VIPN occurs in 30-40% of patients treated with VCR [5, 6].

VIPN is one of the dose-limiting toxicities of VCR, requiring a dose reduction of VCR in the next cycle. Dose reductions of VCR due to VIPN are likely to be related to the treatment outcomes, as dose-dense chemotherapy has been shown to produce better clinical outcomes in the treatment of B-cell lymphoma [7, 8]. The prediction and prevention of VIPN are thus very important in the use of R-CHOP and R-CHOP-like regimens.

It was reported that the total dose of VCR and the number of treatment cycles were related to the incidence of VIPN [9, 10]. However, VIPN will also occur during the first treatment cycle regardless of the total dose of VCR or the number of treatment cycles (namely early-onset VIPN). In their treatment for B-cell lymphoma, patients must undergo chemotherapy repeatedly. If early-onset VIPN develops during the first treatment cycle, the patient will have to continue the treatment while experiencing peripheral neuropathy, and thereby the patient’s quality of life is decreased significantly. It would thus be clinically useful to predict early-onset VIPN before the first cycle of an R-CHOP or R-CHOP-like regimen. However, it is difficult to predict early-onset VIPN because the risk factors for early-onset VIPN have not been identified, and the subset of patients most vulnerable to early-onset VIPN is not known. Moreover, there is little information about early-onset VIPN itself. Here we conducted a retrospective study in patients with early-onset VIPN to identify the risk factors for developing early-onset VIPN.

## Methods

### Study population

This study was reviewed and approved by the Ethics Committee of Tokushima University Hospital.

We analyzed the case records of patients who had their first administration of an R-CHOP or R-CHOP-like regimen in the treatment of B-cell lymphoma between April 2008 and August 2013 at Tokushima University Hospital in Tokushima, Japan. Patients were excluded if they had a history of pre-existing peripheral neuropathy, leaving 41 patients who were eligible for this study. Of the 41 patients, 26 patients had undergone an R-CHOP regimen, five were treated with an R-CVP regimen and 10 had received an R-THP-COP regimen.

In the R-CHOP regimen, rituximab (375 mg/m^2^) was used on day 1, adriamycin (50 mg/m^2^), vincristine (1.4 mg/m^2^, max. 2.0 mg) and cyclophosphamide (750 mg/m^2^) were used on day 2, and prednisolone (100 mg/body) was used from day 2 to day 6. In the R-CVP regimen, rituximab (375 mg/m^2^) was used on day 1, vincristine (1.4 mg/m^2^, max. 2.0 mg) and cyclophosphamide (750 mg/m^2^) were used on day 2, and prednisolone (100 mg/body) was used from day 2 to day 6. In the R-THP-COP regimen, rituximab (375 mg/m^2^) was used on day 1, tetrahydropyranyladriamycin (30 mg/m^2^), vincristine (1.0 mg/m^2^, max. 2.0 mg) and cyclophosphamide (500 mg/m^2^) were used on day 2 and prednisolone (30 mg/m^2^) was used from day 2 to day 6.

### Assessment of risk factors

We collected the data on each patient’s gender, age, body weight, levels of alanine aminotransferase, aspartate aminotransferase, γ-glutamyltranspeptidase, total bilirubin, serum creatinine, lymphoma type, history of diabetes mellitus requiring medication, combined use of the antiemetic aprepitant and the dose of anticancer drugs. The doses of anthracycline drugs were converted to the dose of adriamycin [11]. Creatinine clearance was calculated as described by Cockcroft and Gault [12].

We retrospectively evaluated the presence/absence and degree of peripheral neuropathy in each patient by examining the medical records kept by the hospital’s healthcare workers. We graded adverse events, including peripheral neuropathy, according to the Common Terminology Criteria for Adverse Events v 4.0. We defined early-onset VIPN as grade 1 or higher peripheral neuropathy that occurred during the first cycle of chemotherapy. Based on this measure, we divided the patients into two groups: those with early-onset VIPN (+) and those without early-onset VIPN (-), and we compared the factors between the two groups to identify those associated with early-onset VIPN. The dose of anticancer drugs of the 34 patients who received subsequent chemotherapy in our hospital was also considered in our evaluation of the outcomes of the early-onset VIPN patients.

### Statistical analyses

Fisher’s exact probability test, the Mann-Whitney U test and Student’s *t*-test were used to assess differences between the early-onset VIPN (-) group and early-onset VIPN (+) group. The doses of anticancer drugs at the first cycle of chemotherapy were compared by Student’s *t*-test. Adverse events after the first chemotherapy and the dose adjustment of VCR at next course were assessed by Fisher’s exact probability test. To identify the risk factors for early-onset VIPN, we performed univariate and multivariate logistic regression analyses. The cut-off values for the factors which were significantly different in the univariate analysis were calculated using receiver-operator characteristics (ROC) curves. The sensitivity and specificity of the ROC curves were estimated by determining the area under the ROC curve. In the multivariate analysis, the forced entry method was employed using the factors which were significantly different in the univariate analysis. All analyses were done using Excel (Microsoft). All recorded P values were two-sided, and differences with P values < 0.05 were considered significant.

## Results

### Patient characteristics

Early-onset VIPN was observed in 14 patients (34%): grade 1 early-onset VIPN was observed in 11 patients (79%), grade 2 in two patients (14%) and grade 3 in one (7%). Table 1 shows the patients’ characteristics in the two groups. The patients with early-onset VIPN were significantly younger than the patients without early-onset VIPN (median age: 53 vs. 68 years, P < 0.01). There were no significant differences between the two groups with regard to gender, body weight, liver functions, or renal functions. Lymphoma type and history of diabetes mellitus requiring medication did not differ significantly between the groups.

**Table 1:** Characteristics of the Study Population of 41 Patients Treated With an R-CHOP or R-CHOP-Like Regimen for the First Time

Factor	Early-onset VIPN (-) (n = 27)	Early-onset VIPN (+) (n = 14)	P value
	Means ± SD or no. of patients (%)	Means ± SD or no. of patients (%)	
Peripheral neuropathy			
Grade 1		11 (79)	
Grade 2		2 (14)	
Grade 3		1 (7)	
Grade 4		0 (0)	
Gender (male/female)	16/11	5/9	0.20^a^
Age, median (range) (years)	68 (41 - 87)	53 (34 - 72)	0.0064^b^
Body weight	59.9 ± 10.4	56.2 ± 11.3	0.31^c^
Aspartate aminotransferase	28.9 ± 12.4	22.0 ± 11.6	0.59^c^
Alanine aminotransferase	15.5 ± 8.1	25.3 ± 13.7	0.12^c^
γ-glutamyl transpeptidase	33.8 ± 24.9	48.5 ± 34.7	0.57^c^
Total bilirubin	0.60 ± 0.24	0.67 ± 0.24	0.37^c^
Creatinine clearance	97.9 ± 40.3	112.9 ± 18.5	0.20^c^
Diabetes mellitus requiring medication	4 (15)	1 (7)	0.66^a^
Lymphoma type			
Diffuse large B-cell lymphoma	15 (56)	9 (64)	0.74^a^
Follicular lymphoma	7 (26)	2 (14)	0.70^a^
Others	5 (18)	3 (22)	1.00^a^
Regimen			
Normal-dose chemotherapy	17 (63)	14 (100)	0.0085^a^
R-CHOP	14 (52)	12 (86)	0.044^a^
R-CVP	3 (11)	2 (14)	1.00^a^
Reduced-dose chemotherapy			
R-THP-COP	10 (37)	0 (0)	0.0085^a^
Concomitant use of aprepitant	9 (33)	10 (71)	0.026^a^

^a^Fisher’s exact probability test. ^b^Mann-Whitney U test. ^c^Student’s *t*-test.

**Figure 1:**
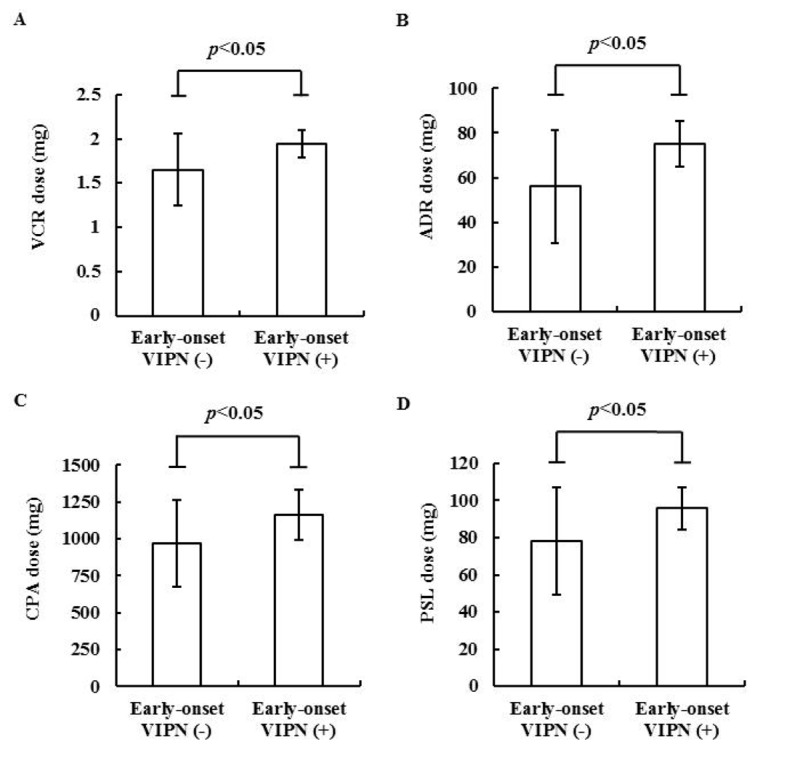
The doses of VCR (A), adriamycin (B), cyclophosphamide (C) and prednisolone (D) in the early-onset VIPN (-) group (n = 27) and the early-onset VIPN (+) group (n = 14). ADR: adriamycin; CPA: cyclophosphamide; PSL: prednisolone.

The population of patients treated with an R-CHOP or R-CVP regimen for whom the normal dose of anticancer drugs was administered was significantly greater among the patients with early-onset VIPN (100% vs. 63%, P < 0.01), whereas the percentage of R-THP-COP-treated patients for whom a reduced dose of anticancer drugs was administered was significantly greater in the group without early-onset VIPN (0% vs. 37%, P < 0.01). This result reflected the finding that the doses of anticancer drugs in the early-onset VIPN (+) group were greater than those in the early-onset VIPN (-) group (Fig. 1).

The population of patients concomitantly using aprepitant was significantly greater among those with early-onset VIPN (71% vs. 33 %, P < 0.05). The percentage of patients who experienced adverse events of chemotherapy other than peripheral neuropathy was not significantly different between the two groups (Table 2). We assessed the dose of VCR at the subsequent chemotherapy in the 34 patients who received subsequent chemotherapy at our hospital (early-onset VIPN (+): n = 10, early-onset VIPN (-): n = 24) to evaluate the outcomes for early-onset VIPN. Although there were no patients in the early-onset VIPN (-) group who had received a reduced VCR dose at the subsequent cycle of chemotherapy, in the early-onset VIPN (+) group one patient received a reduced VCR dose and one patient discontinued the administration of VCR at the next chemotherapy (Table 3).

**Table 2:** Adverse Events After the First Cycle of Chemotherapy

Adverse event	Early-onset VIPN (-) (n = 27)	Early-onset VIPN (+) (n = 14)	P value
	No. of patients (%)	No. of patients (%)	
Neutropenia (≥ grade 3)	17 (63)	12 (86)	0.16
Febrile neutropenia	3 (11)	1 (7)	1.00
Thrombocytopenia (≥ grade 3)	1 (4)	0 (0)	1.00
Constipation	8 (30)	4 (29)	1.00
Nausea	5 (19)	5 (36)	0.27
Vomiting	1 (4)	1 (7)	1.00

**Table 3:** Dose Modifications of VCR in Patients Who Received Subsequent Chemotherapy at Our Hospital

Factor	Early-onset VIPN (-) (n = 24)	Early-onset VIPN (+) (n = 10)	P value
	No. of patients (%)	No. of patients (%)	
VCR dose modifications at next cycle			
Unmodified	24 (100)	8 (80)	0.080
Modified	0 (0)	2 (20)	
Discontinued	0 (0)	1 (10)	
Reduced	0 (0)	1 (10)	

### Univariate analysis

**Figure 2:**
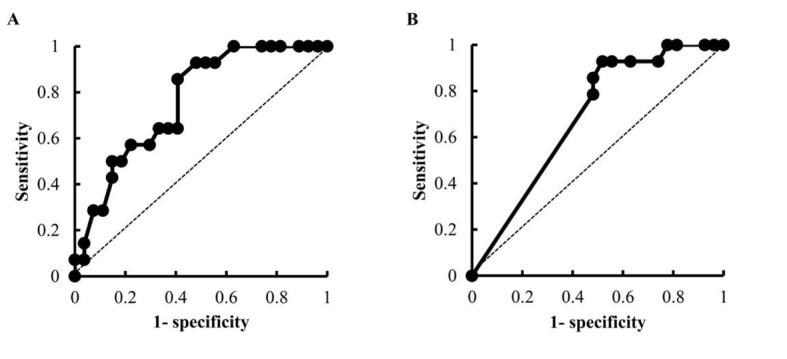
ROC curves of age (A) and the dose of VCR (B) obtained by univariate logistic regression analysis.

To identify the risk factors for the development of early-onset VIPN, we analyzed the association between the occurrence of early-onset VIPN and patient characteristics by performing a univariate logistic regression analysis. The analysis revealed significant correlations between the incidence of early-onset VIPN and patient age, the dose of VCR, and the concomitant use of aprepitant (Table 4). To estimate the prediction ability of early-onset VIPN, we calculated the area under the ROC curves (AUC) obtained by the univariate analysis for age and the dose of VCR. The AUC values were 0.76 and 0.69, respectively, which was evaluated as having moderate accuracy for detecting some events (Fig. 2). The cut-off values of age and the dose of VCR calculated by the ROC curve were 65 years and 1.9 mg, respectively.

**Table 4:** Univariate Logistic Regression Analysis to Identify the Risk Factors for Early-Onset VIPN

Factor	Odds ratio	95% CI	P value
Male	0.38	0.10 - 1.45	0.16
Age	0.92	0.86 - 0.98	0.013*
Body weight	0.97	0.91 - 1.03	0.31
Aspartate aminotransferase	1.01	0.96 - 1.07	0.59
Alanine aminotransferase	1.05	0.98 - 1.12	0.14
γ-glutamyltranspeptidase	1.00	0.98 - 1.03	0.56
Total bilirubin	3.41	0.25 - 47.00	0.36
Creatinine clearance	1.01	0.99 - 1.03	0.21
Diabetes mellitus requiring medication	0.44	0.045 - 4.39	0.49
Lymphoma type			
Diffuse large B-cell lymphoma	1.44	0.38 - 5.45	0.59
Follicular lymphoma	0.48	0.085 - 2.68	0.40
Others	1.20	0.24 - 5.97	0.80
VCR dose	40.81	1.09 - 1,524.04	0.045*
Concomitant use of aprepitant	5.00	1.22 - 20.46	0.025*

*Included in the multivariate logistic regression analysis.

### Multivariate analysis

We carried out a multivariate logistic regression analysis using the factors that were significantly different in the univariate analysis. The results of the multivariate analysis (Table 5) revealed that the patients who had received a VCR dose ≥ 1.9 mg (odds ratio (OR) 15.29; 95% confidence interval (CI) 1.14 - 205.66; P < 0.05) or had a concomitant use of aprepitant (OR 6.09; 95% CI 1.15 - 32.11; P < 0.05) had a significantly higher risk of developing early-onset VIPN. Therefore, we chose the factors of VCR dose ≥ 1.9 mg and a concomitant use of aprepitant as independent risk factors for early-onset VIPN.

**Table 5:** Risk Factors for Developing Early-Onset VIPN by Multivariate Logistic Regression Analysis

Factor	Odds ratio	95% CI	P value
Age ≥ 65 years	0.90	0.13 - 6.38	0.92
VCR dose ≥ 1.9 mg	15.29	1.14 - 205.66	0.039
Concomitant use of aprepitant	6.09	1.15 - 32.11	0.033

## Discussion

VCR acts by inhibiting the assembly and promoting the disassembly of microtubules. VCR also interferes with axonal transport, causing peripheral neuropathy [13]. It was reported that 30-40% of patients treated with VCR develop VIPN [5]. In the present study, 14 patients (34%) had grade 1 or higher VIPN after their first chemotherapy. We may find that VIPN is more frequent if we continue observing the patients in our study, but we suspect that there will not be very different results regarding the incidence of VIPN between the present study and previous reports. Moreover, a previous study reported that the incidence of grade 2 or higher early-onset VIPN was 4% [14]. In our study, grade 2 or higher early-onset VIPN was observed in only three patients (7.3%), showing the near equivalence of the incidence of early-onset VIPN in our study compared to previous reports.

Grade 2 or higher peripheral neuropathy due to anticancer drugs causes a decrease in a patient’s quality of life [15]. Analyses of patients with grade 2 or higher peripheral neuropathy will be useful to identify the risk factors associated with severe early-onset VIPN, but we defined early-onset VIPN as grade 1 or higher peripheral neuropathy due to the low number of patients who had grade 2 or higher VIPN in the present study. It is important to be able to predict early-onset VIPN including grade 1 VIPN because grade 1 VIPN may progress to grade 2 or higher during the repeated chemotherapy in the treatment of B-cell lymphoma.

Our multivariate logistic regression analysis revealed that the patients who received a VCR dose ≥ 1.9 mg appeared to have a higher risk of early-onset VIPN. Previous studies showed that the total dose of VCR and the number of treatment cycles were related to the incidence of VIPN [9, 10]. Our present findings revealed that a single dose of VCR was also a risk factor for early-onset VIPN. In the R-CHOP or R-CVP regimens, the full dose of VCR is 2.0 mg. Therefore, the full dose of VCR can be described as a risk factor for early-onset VIPN. Indeed, 13 patients (92.9%) in the present study’s early-onset VIPN (+) group received the full dose of VCR. On the other hand, 14 patients (51.9%) in the early-onset VIPN (-) group received the reduced dose of VCR. We should therefore pay special attention to the possibility of the development of early-onset VIPN when the full dose of VCR is administered.

The metabolism of VCR has been shown to be mediated by CYP3A4, and previous studies showed that CYP3A4 inhibitors increase the therapeutic concentration of VCR and cause VIPN [16-18]. Our multivariate logistic regression analysis revealed that the patients who had a concomitant use of aprepitant appeared to have a higher risk of early-onset VIPN. Aprepitant, a neurokinin_1_ receptor antagonist, is known to be an inhibitor of CYP3A4 [19]. It was also shown that the AUC values of dexamethasone and methylprednisolone, which are metabolized by CYP3A4, are increased by the concomitant use of aprepitant [20]. We therefore speculate that the concomitant use of aprepitant increases the therapeutic concentration of VCR by inhibiting CYP3A4, causing early-onset VIPN. However, a prospective study will be needed to elucidate the relationship between VCR concentration and the effect of CYP3A4 inhibition of aprepitant; there is as yet no available data regarding the pharmacokinetics and pharmacodynamics of VCR with the concomitant use of aprepitant.

Aprepitant shows an antiemetic effect in various chemotherapies [21, 22], but there is little information regarding aprepitant’s antiemetic effect in patients treated with an R-CHOP or R-CHOP-like regimen. In the present study, there were no significant differences in the rates of nausea and vomiting between the early-onset VIPN (-) and early-onset VIPN (+) groups despite the high concomitant use of aprepitant in the early-onset VIPN (+) group (Table 2). This may be due to the higher dose of anticancer drugs in the early-onset VIPN (+) group (Fig. 1). Therefore, it is important to use aprepitant in light of the risk of early-onset VIPN and the benefit of aprepitant’s antiemetic effect in R-CHOP and R-CHOP-like regimens.

According to our univariate analysis, age was a significant candidate as a risk factor for early-onset VIPN, but the significance disappeared in the multivariate analysis. There was a significant negative correlation between age and the dose of VCR in our study population (Pearson r = -0.69, P < 0.0001). This is because age is one of the administration criteria for the R-THP-COP regimen. We hypothesize that age was not identified as a risk factor for early-onset VIPN due to the high correlation between age and the dose of VCR. On the other hand, there was no correlation between the dose of VCR and the concomitant use of aprepitant, which was identified as a risk factor for early-onset VIPN (data not shown). Thus, no evidence of multicollinearity among independent variables was found.

Several research groups have attempted to clarify the relationship between genetic factors and the metabolism of VCR [23]. Broyl et al reported that early-onset VIPN was associated with the presence of single-nucleotide polymorphisms (SNPs) in genes involved in the absorption, distribution, metabolism and excretion of VCR [14]. The presence of SNPs of CYP3A4 has been described [24], this may be associated with early-onset VIPN. Moreover, it is reported that pregabalin and gabapentin are effective for the treatment of VIPN [25, 26]. In a murine model, lithium and the antiemetic tropisetron were also reported to be effective for VIPN [27, 28]. In our study, one patient with grade 3 early-onset VIPN had received pregabalin, but the other patients had received no medication for VIPN. Further studies are needed to elucidate the SNPs associated with early-onset VIPN and the effectiveness of drug therapies for early-onset VIPN.

In conclusion, the results of the present study showed that the dose of VCR and the concomitant use of aprepitant were independent risk factors for early-onset VIPN. Although further prospective analyses with more patients are needed to collect further evidence, the present findings provide useful information regarding the monitoring of early-onset VIPN.
